# Emerging role of mesenchymal stromal cells (MSCs)-derived exosome in neurodegeneration-associated conditions: a groundbreaking cell-free approach

**DOI:** 10.1186/s13287-022-03122-5

**Published:** 2022-08-19

**Authors:** Hadi Yari, Maria V. Mikhailova, Mahsa Mardasi, Mohsen Jafarzadehgharehziaaddin, Somayeh Shahrokh, Lakshmi Thangavelu, Hosein Ahmadi, Navid Shomali, Yoda Yaghoubi, Majid Zamani, Morteza Akbari, Samira Alesaeidi

**Affiliations:** 1 Medical Biotechnology Department, National Institute of Genetics Engineering and Biotechnology (NIGEB), Tehran, Iran; 2 Department of Prosthetic Dentistry, Sechenov First Moscow State Medical University, Moscow, Russia; 3 Biotechnology Department, Faculty of Life Sciences and Biotechnology, Shahid Beheshti University G. C, Evin, Tehran, Iran; 4 Translational Neuropsychology Lab, Department of Education and Psychology and William James Center for Research (WJCR), University of Aveiro, Campus Universitário de Santiago, 3810-193 Aveiro, Portugal; 5 Department of Pathobiology, Faculty of Veterinary Medicine, University of Shahrekord, Shahrekord, Iran; 6 Department of Pharmacology, Saveetha Dental College, Saveetha Institute of Medical and Technical Science, Saveetha University, Chennai, India; 7 Department of Molecular Medicine, Institute of Medical Biotechnology, National Institute of Genetic Engineering and Biotechnology (NIGEB), Tehran, Iran; 8 Immunology Research Center, Tabriz University of Medical Sciences, Tabriz, Iran; 9 School of Paramedical, Kermanshah University of Medical Sciences, Kermanshah, Iran; 10 Department of Medical Laboratory Sciences, Faculty of Allied Medicine, Infectious Diseases Research Center, Gonabad University of Medical Sciences, Gonabad, Iran; 11 Department of Internal Medicine and Rheumatology, Rheumatology Research Center, Tehran University of Medical Sciences, Tehran, Iran

**Keywords:** Neurodegeneration, Mesenchymal stromal cells (MSCs), Exosome, Neurogenesis, Neuroinflammation

## Abstract

Accumulating proofs signify that pleiotropic effects of mesenchymal stromal cells (MSCs) are not allied to their differentiation competencies but rather are mediated mainly by the releases of soluble paracrine mediators, making them a reasonable therapeutic option to enable damaged tissue repair. Due to their unique immunomodulatory and regenerative attributes, the MSC-derived exosomes hold great potential to treat neurodegeneration-associated neurological diseases. Exosome treatment circumvents drawbacks regarding the direct administration of MSCs, such as tumor formation or reduced infiltration and migration to brain tissue. Noteworthy, MSCs-derived exosomes can cross the blood–brain barrier (BBB) and then efficiently deliver their cargo (e.g., protein, miRNAs, lipid, and mRNA) to damaged brain tissue. These biomolecules influence various biological processes (e.g., survival, proliferation, migration, etc.) in neurons, oligodendrocytes, and astrocytes. Various studies have shown that the systemic or local administration of MSCs-derived exosome could lead to the favored outcome in animals with neurodegeneration-associated disease mainly by supporting BBB integrity, eliciting pro-angiogenic effects, attenuating neuroinflammation, and promoting neurogenesis in vivo. In the present review, we will deliver an overview of the therapeutic benefits of MSCs-derived exosome therapy to ameliorate the pathological symptoms of acute and chronic neurodegenerative disease. Also, the underlying mechanism behind these favored effects has been elucidated.

## Introduction

Neurodegenerative conditions are heterogeneous disorders characterized primarily by the progressive loss of neurons in the brain or spinal cord [1]. During acute neurodegeneration, neurons are promptly damaged and then destructed in response to a sudden insult (e.g., trauma) [2]. Chronic neurodegeneration develops over a prolonged period, causing the loss of a particular neuronal subtype [3]. Indeed, acute neurodegeneration is found in conditions such as spinal cord injury (SCI), traumatic brain injury (TBI), and stroke. Besides, chronic neurodegeneration is shown in Alzheimer’s disease (AD), Parkinson’s disease (PD), Huntington’s disease (HD), amyotrophic lateral sclerosis (ALS), and multiple sclerosis (MS) [4, 5]. Given the diverse and multifaceted mechanisms of neuronal loss, finding or designing an efficient and practical therapeutic strategy is challenging. However, it seems that neuroinflammation plays a critical role in their pathogenesis because of the presence of inflammatory mediators at high levels in the brain tissue of rodents with neurodegeneration [6, 7]. Thus, targeting neuroinflammation to induce a neuroprotective effect and stimulating neurogenesis to substitute destructed neurons is a rational therapeutic plan to provide preferred therapeutic outcomes in vivo.

**Fig. 1:**
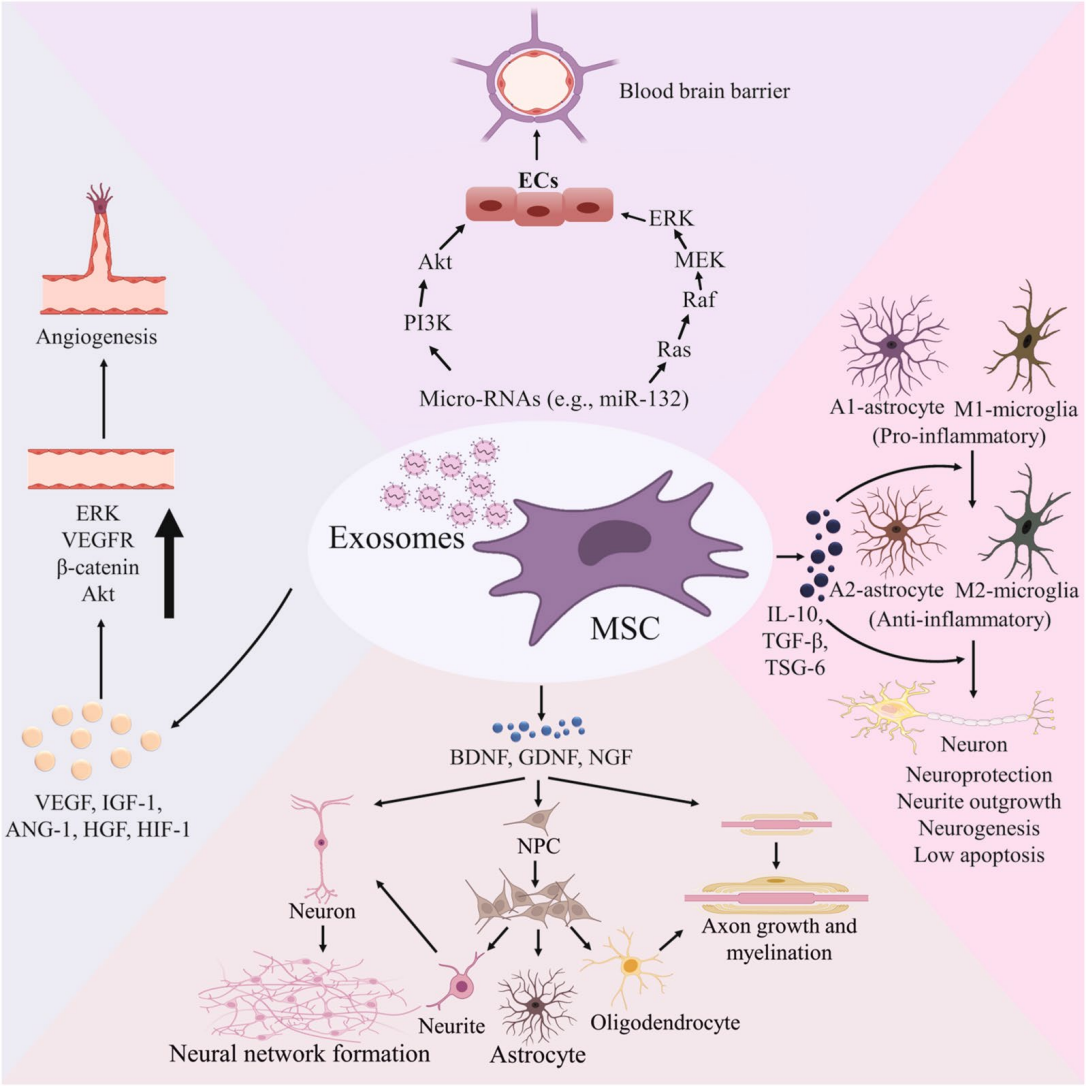
Underlying mechanisms behind the mesenchymal stromal cell (MSCs)-derived exosome-mediated favored effects on neurodegeneration-associated diseases. Due to the presence of growth factor, miRNAs, and anti-inflammatory mediators, exosome treatment induces angiogenesis and neurogenesis, improves blood–brain barrier (BBB) integrity, and also attenuates neuroinflammation

Since the 1980s, when stem cell therapy on PD patients exhibited inspiring outcomes [8], stem cell technology has been considered a potential therapeutic modality in the context of neurological disease therapy. In this light, mesenchymal stromal cells (MSCs)-based therapeutics have attracted increasing attention because of their isolation from various adult tissue, easy ex vivo expansion, and also low immunogenicity. These properties make them an ideal source in either an autologous or allogeneic manner [9, 10]. They are capable of differentiation into neural cell lineages, such as neurons, astrocytes, and oligodendrocytes (ODCs) [11–13]; however, it seems that the MSCs-mediated positive effect mainly depends on their paracrine effects rather than their direct differentiation potential [14, 15]. Among them, inducing neuroprotection, neurogenesis, and angiogenesis, inhibiting neuroinflammation, promoting blood–brain barrier (BBB) integrity, and degradation of aberrant protein aggregates are of paramount significance [16, 17]. These effects are mostly elicited by secretion of neurotrophic factors (NTFs), such as glial cell-derived neurotrophic factor (GDNF), nerve growth factor (NGF), and brain-derived neurotrophic factor (BDNF), and also producing anti-inflammatory mediators like transforming growth factor-β (TGF-β) and interleukin-10 (IL-10), and tumor necrosis factor alpha-stimulated gene-6 (TSG-6) (Fig. 1) [1, 18].

During the last decades, researchers have sought different strategies to improve the therapeutic effects of MSCs. Accordingly, MSCs-derived exosomes have become a promising novel cell-free approach. MSCs-derived exosomes bypass difficulties related to the MSC’s direct use, such as aging, possible tumor formation, and low engraftment, to target tissue due to the presence of BBB [19]. Exosomes are nano-sized, lipid bilayer-enclosed structures, which are secreted from various cells, particularly stem cells, immune cells, and tumor cells [20, 21]. MSCs-derived exosomes can cross the BBB and exert robust and prolonged neuroprotection and neurogenesis [22, 23]. Indeed, they are hypoimmunogenic nanocarriers and comprise numerous immunoregulatory, neurotrophic, pro-survival, and pro-angiogenic mediators [24–26]. Thus, exosomes exhibit a unique capability to ameliorate neurodegenerative disease-associated deficits [27, 28]. Herein, we have focused on the therapeutic application of MSCs-derived exosome as an emerging and rapidly evolving cell-free therapeutic approach to alleviate neurodegeneration and stimulate neurogenesis, with particular emphasis on last decade’s in vivo reports.

## The rationality of MSCs-derived exosome therapy in neurodegeneration-related conditions

### Suppression of neuroinflammation

Neuroinflammation is a multifaceted process in which a spectrum of inflammatory responses emerges and ultimately leads to neural cell loss [29]. Deregulated microglia and astrocytes activation accompanied by up-regulated levels of the pro-inflammatory molecules is commonly observed in patients suffering from MS, PD, ALS, HD, AD, SCI, TBI, and also stroke [30]. Neuroinflammation also abrogates the endogenous brain repair process, thus impairing neural tissue recovery [31]. Microglia and astrocytes are induced into two polarization statuses during neuroinflammation: pro-inflammatory phenotype (M1 and A1) and the anti-inflammatory phenotype (M2 and A2). Microglia (M1)-mediated inflammatory responses largely contribute to various neurological diseases associated with neural damage [32]. M1 activation of microglia is described as an undesired event and is complicated in the development of neurological disease. M1 microglia induces immediate inflammation by secretion of pro-inflammatory cytokines, such as IL-1, IL-6, and TNF-ɑ. They also regulate the function and status of neurons, astrocytes, and ODCs [33]. Pro-inflammatory cytokines also have a correlation with enhanced numbers of A1 reactive astrocytes in the damaged tissue. Besides, M2 activation of microglia leads to the secretion of anti-inflammatory mediators, such as IL-10, TGFβ, and glucocorticoids [34]. Aberrant activation of the NLR family pyrin domain containing 3 (NLRP3) inflammasome, a crucial part of the innate immune system, also contributes to the development of various neurodegenerative conditions, including AD and PD [35, 36]. Inflammatory brain responses are also related to the up-regulated prostaglandins (PGs) levels, particularly PGE2 [6].

A large number of studies have shown that MSCs can down-regulate M1 microglia and A1 astrocyte activation, thus inducing neuroprotective effects [37, 38]. In vitro results indicated that MSCs’ co-culture with amyloid beta-peptide (Aβ)-induced neural cells resulted in the release of IL-10 and TGF-β into the culture medium [39]. The shift from a pro-inflammatory to an anti-inflammatory environment was also found in the lumbar spinal cord of ALS mice following umbilical cord (UC)-MSCs therapy [40]. Likewise, MSCs-derived exosomes elicited strong anti-inflammatory influences in a subarachnoid hemorrhage (SAH) [41] and AD rodent model by improving M2-polarized macrophages numbers [42]. MSCs also inhibit inflammatory response by up-regulation of TSG-6 [43, 44] and down-regulation of NLRP3 expression [45, 46], which obstructs microglia activation as shown in TBI and SCI animal models. In the ALS mice model, results also revealed that MSCs-derived exosome administration by intramuscular [47] and intraventricular [40] routes impaired disease development and reduced the inducible nitric oxide (NO) synthase (iNOS) activation and subsequent NO syntheses [47]. In sum, MSCs' robust anti-inflammatory and immunomodulatory effects justify their application in neurological diseases associated with neuroinflammation.

### Neurotrophic factors (NTFs) release

Neurotrophic factors (e.g., BDNF, NGF, and GDNF) are endogenous biomolecules mainly contributing to cell proliferation and differentiation in the nervous system. They are also implicated in synaptic plasticity and long-term memories [45]. Based on findings, changes in the levels of neurotrophic factors or their receptors are thought to be responsible for neuronal deterioration and also participate in neurodegenerative diseases’ pathogenesis [46, 48, 49]. Apart from anti-inflammatory action, functional rescue after MSCs therapy mainly arises from neurotrophic factors delivery to brain tissue, thus provoking neuroprotection and neurogenesis [50–54]. The existence of the NTFs such as BDNF, NGF, and GDNF in MSCs-derived exosomes has strongly been exhibited by molecular analysis [55, 56].

In rodent models of AD, results exhibited that UC‐MSCs injection by intrahippocampal [50] and intrathecal [51] route led to cognitive deficits rescue and also facilitated neural networks formation by secretion of hepatocyte growth factor (HGF) [50] and growth/differentiation factor-15 (GDF-15) [51]. In the hippocampus of experimental models, HGF and GDF-15 induce signaling pathways involved in neural cell survival, proliferation, and migration [57]. Further, systemic injection of AT-MSC brought about the up-regulation of dopamine transporter expression and inspired functional rescue in PD rodent models due to BDNF and GDNF delivery [52]. In addition, Ebrahimi et al. (2018) demonstrated that GDNF and vascular endothelial growth factor (VEGF) secretion by MSCs favored motor coordination and muscle functions in HD animal models [58].

Irrespective of the inherent potential of MSCs to secret NTFs, genetically modified MSCs to overexpress NTFs provide a promising therapeutic approach to treating neurological diseases. Meanwhile, BDNF-overexpressing MSCs elicited appreciated in vivo outcomes upon transplantation into a PD monkey model [59], SCI rat model [60], and also mice model of HD [61] and ALS [62]. Based on the in vivo reports, BDNF-overexpressing MSCs could restore motor function and improve overall survival (OS) in treated animals. Besides, GDNF-overexpressing MSCs could reduce neuroinflammation and consequently down-regulate neurodegeneration in the rat models of PD [63] and ALS [64]. There are also five ongoing or completed registered trials designed to address the safety and efficacy of intramuscular and intrathecal administration of NurOwn^®^ (MSC-NTF) in patients with ALS (NCT02017912, NCT04681118, NCT01777646, and NCT01051882) and MS (NCT03799718). Meanwhile, Berry and colleagues (2019) findings [65] verified the safety and efficacy of single-dose intrathecal and intramuscular transplantation of MSC-NTF in ALS patients. They showed improved levels of the neurotrophic factors in cerebrospinal fluid (CSF) in ALS patients. At the same time, CSF inflammatory biomarkers were reduced in treated patients, highlighting the central roles of immunomodulation and NTFs delivery in MSCs-mediated therapeutic influences in vivo [65].

### Stimulating angiogenesis

Angiogenesis, the growth of new blood vessels, is a natural defense mechanism helping to restore oxygen and nutrient supply to the damaged brain tissue upon ischemia or similar conditions. By stimulating vessel growth, angiogenesis may stabilize brain perfusion and potentiate neuronal survival, brain plasticity, and neurologic recovery [66]. During neurodegeneration, the neural progenitor cells’ (NPCs) migration to regenerate damaged neurons is facilitated by blood vessels. Thereby, it was hypothesized that angiogenesis might provide succeeding neurogenesis [67, 68]. Interestingly, up-regulated levels of angiogenic growth factors such as VEGF and its receptors are shown in brain tissue after several neurological diseases, such as TBI and stroke [69]. In addition to the activating signaling pathways involved in angiogenesis, MSCs-derived exosomes deliver pro-angiogenic factors such as VEGF, EGF, and FGF-1 directly to the target tissue [70].

In 2017, Hung and coworkers showed that intravenous injection of MSCs-derived exosome stimulated angiogenesis in SCI rats, while the underlying mechanism was not elucidated [71]. However, other reports have shown that deposition of fibronectin (FN) accompanied by up-regulation of the expression of VEGF, HGF, insulin-like growth factor 1 (IGF-1), angiopoietin-1 (Ang-1), and hypoxia-inducible factor 1-alpha (HIF-1α) contributes to inducing angiogenesis upon MSCs’ therapy. These biomolecules transduce various signaling axes, particularly phosphatidylinositol-3-kinase (PI3K)/Akt pathway [72–74]. For example, perivascular delivery of encapsulated MSCs enhanced postischemic angiogenesis by paracrine induction of VEGF-A [75]. Likewise, BM-MSCs’ transplantation potentiated VEGF and ANG-1 expressions and, in turn, enhanced the formation of microvessels in brain tissues after TBI in mice models [76]. Also, HGF-overexpressing BM-MSCs’ therapy promoted angiogenesis in an ischemic rat [77]. Hypoxic preconditioning of MSCs resulted in the up-regulation of HIF-1α, VEGF, erythropoietin (EPO), stromal-derived factor-1 (SDF-1), and C-X-C chemokine receptor type 4 (CXCR4), thus potentiating their pro-angiogenic capacities in stroke rodents [78]. MSCs also underlie neurogenesis and repair neural damage by stimulating endogenous angiogenesis and up-regulation of angiogenic mediators secreted from activated astrocytes [79].

### Others

In the brain, the vascular endothelium acts as a critical part of the BBB due to its suitable construction to provide a functional and molecular dissociation of the brain from the rest of the body and defend neurons versus pathogens and toxins [80]. Thus, deregulated transportation of metabolites across the BBB because of its dysfunction might elicit adverse effects on brain health and cognitive function [81, 82]. Indeed, the BBB damage contributes to impaired peripheral–CNS interaction, thereby provoking neurodegeneration [83]. Besides, because of the significant vulnerability of the brain to oxidative damage and high levels of ROS, mitigation of oxidative stress is urgently required to enable efficient treatments of neurodegeneration [84–87]. Although low levels of ROS play physiological roles in cell signaling, various clinical trials based on targeting increased levels of ROS are ongoing using antioxidant agents.

Recent reports have indicated that MSCs-derived exosomes could transfer a myriad of microRNAs (miRs), such as miR-132-3p, to endothelial cells (ECs), which in turn improves their proliferation and thus alleviates BBB impairment [88]. A study in the middle cerebral artery occlusion (MCAO) mouse model exhibited that miR-132-3p could inhibit RASA1, while improving Ras and PI3K phosphorylation [88]. In addition to these effects, miR-132-3p-enriched exosome reduced ROS production and ECs apoptosis and supported tight junctions. This study offers clear evidence signifying the positive effect of exosome therapy on BBB integrity and also eliciting antioxidant effects in vivo [88]. Likewise, exosome treatment also significantly enhanced the expression of genes involved in promoting the BBB stability, including claudin-5, occludin, tight junction protein 1 (TJP1), laminin subunit B1 (LAMB), and RUNX family transcription factor 1 (RUNX1) in TBI rodent models [89]. Additionally, Williams et al. (2020) showed that exosome-treated animals had reduced albumin extravasation and higher laminin, claudin-5, and zonula occludens 1 (ZO1) levels [90]. These events ultimately diminished brain swelling and lesion size, down-regulated blood-based cerebral biomarkers, and finally enhanced BBB integrity [90].

Furthermore, Katsuda et al. (2013), for the first time, demonstrated that human AT-MSCs secrete exosomes carrying enzymatically active neprilysin (NEP) [91]. The NEP is the most significant amyloid-β (Aβ)-degrading enzyme in the brain. They suggested that NEP-enriched exosome could attenuate both secreted and intracellular Aβ levels in neural cells [91]. Besides, MSCs-derived exosomes could reduce the Aβ levels and promote the expression of NEP in APP/PS1 [92]. Of course, further evidence is required to ascertain the putative capacity of MSCs-derived exosome to degrade Aβ.

## Exosome therapy as a cell-free approach

Exosomes are a subtype of extracellular vesicles (EVs) with a diameter in the range of 30–100 nm. They are usually released by various human and animal cells such as stem cells [93]. Exosomes include multiple biomolecules, including proteins, lipids, messenger RNA (mRNA), and most importantly, microRNAs (miRNAs) as cargo. Exosomes are secreted in a firmly regulated process: formation of endocytic vesicles by invagination of the plasma membrane, generation of multivesicular bodies (MVBs) upon endosomal membranes’ inward budding, and finally merging of shaped MVBs with the plasma membrane and release of the vesicular contents termed exosome [94]. Brain tissue recovery and functional rescue in neurological diseases upon MSCs therapy are chiefly due to the MSCs-mediated paracrine effect. This fact confirms the importance of MSCs-derived exosome therapy rather than direct MSCs’ transplantation. Indeed, MSCs-derived exosome targets biological processes in recipient cells while alleviating comprehensions concerning the immediate use of parental cells (e.g., aging and tumor formation) [95]. Overall, such vesicles show better stability in circulation, improved biocompatibility, low immunogenicity, and toxicity compared with parental stromal cells [96].

The inability of most drugs to cross the BBB is the main problem of modern neuropharmacology. Exosomes reproduce a critical benefit in this light since they can cross BBB [97]. Accumulating information offered by animal studies has shown that MSC-derived exosomes can re-induce self-tolerance without complications observed during the direct MSCs transplantation. Given that the exosome cargos rely on their cellular origin, the composition of MSCs-derived exosomes is similar to that of parental cells. They express common surface markers and membrane-binding proteins, such as CD73, CD44, and CD29 [98]. As cited, exosomes can attenuate neuroinflammation, inspire neurogenesis and angiogenesis, restore spatial learning deficits, and support functional recovery in neurological disease [99–102]. MSCs-derived exosomes can up-regulate the levels of anti-inflammatory molecules and down-regulate pro-inflammatory molecules levels. They suppress macrophage activation by inhibition of Toll-like receptor (TLR) signaling and also reduce hypoxic inflammation by down-regulation of pro-proliferative pathways, such as signal transducer and activator of transcription 3 (STAT3) phosphorylation [103].

**Fig. 2:**
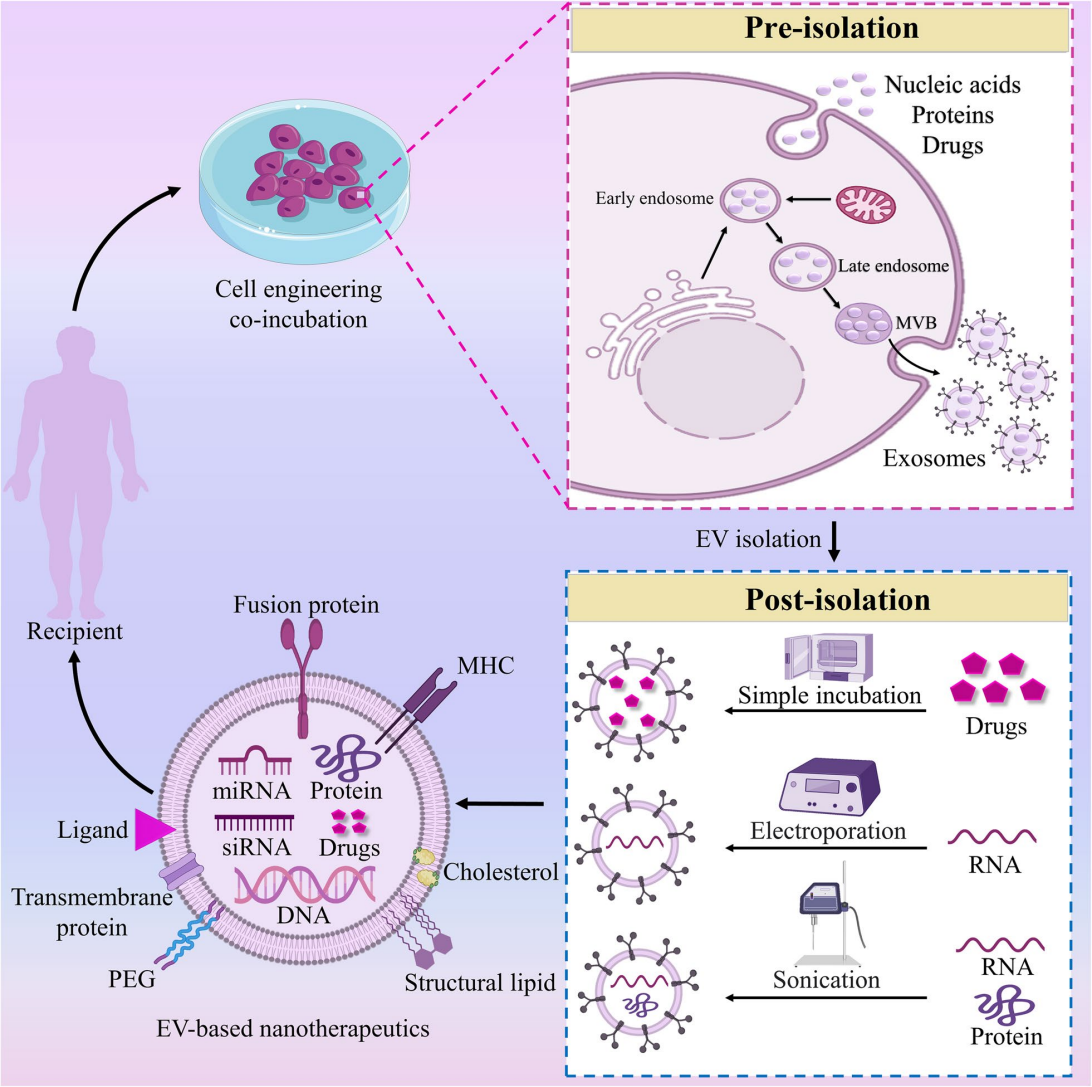
Potentiating the therapeutic capacity of the mesenchymal stromal cell (MSCs)-derived exosome. To promote the efficacy of exosome therapy, parental cells’ modification (pre-isolation) or manipulation of exosome (post-isolation) are being used in preclinical studies

Meanwhile, other results revealed that MSCs-derived exosomes could augment the number of newborn neurons detected in the granule layer of the dentate gyrus (DG) of the hippocampus in TBI animal models [104]. Significantly, various approaches are developed to improve the therapeutic capabilities of exosomes. The current methods are concentrated on two main strategies: cellular modification by preconditioning, such as genetic modification and pre-treatment (pre-isolation), and manipulation of isolated exosome (post-isolation) (Fig. 2). For instance, Xin et al. (2012) showed that co-culture of MSCs with brain tissue extracted from rats with ischemic stroke might improve the quantity of miR-133b in MSCs-derived exosomes [105]. MSCs’ ischemic preconditioning also may result in promoted levels of miR-22 in isolated exosomes [106]. These miRNAs promote neurite remodeling, potentiate ECs proliferation, and improve functional recovery in rodents with neurological disease [107, 108]. In addition, loading the MSCs-derived exosome with curcumin upon isolation and before intranasal transplantation improved the movement and coordination ability of the PD mice model, based on Peng et al. (2022) reports [109]. Curcumin can excite developmental and adult hippocampal neurogenesis and reinforce neural plasticity and repair [110].

## MSCs-derived exosome in acute neurodegeneration

### SCI

Spinal cord injury (SCI) is characterized by temporary or permanent changes in spinal cord function due to spinal cord damage with traumatic and non-traumatic etiologies [111]. During SCI, damage to neurons and ODCs impairs their normal functions and disrupts vasculature and the blood–spinal cord barrier, leading to neurological dysfunction [112]. Destruction of spinal cord microvascular supply leads to cell permeabilization, pro-apoptotic signaling, and ischemic injury [113]. The recruitment of inflammatory cells, cytokines, and vasoactive peptides, as shown by up-regulated levels of the TNF-ɑ and IL-1β, is demonstrated within minutes of injury [114, 115].

During neurodegeneration, A1 astrocytes lose their normal astrocytic function while acquiring a new neurotoxic action, thereby killing neurons and mature ODCs [116]. Recent results revealed that BM-MSCs-derived exosomes could display pro-angiogenic properties, attenuate glial scar formation, decrease lesion volume, augment axonal regeneration, and also improve functional recovery. These effects are mainly mediated by various mechanisms such as down-regulation of nitric oxide (NO) release in microglia in association with inhibition of the activation of A1 neurotoxic reactive astrocytes [117]. MSC-derived exosomes may also diminish SCI-induced A1 astrocytes potently by down-regulation of the nuclear translocation of NFκB p65, thereby inspiring anti-inflammatory and neuroprotective effects on SCI animal models [118]. M1 macrophages are implicated in further damage to the spinal cord, whereas M2 macrophages trigger regenerative growth responses in adult sensory axons [119]. Recently, Liu et al. (2020) showed that miR-216a-5p-enriched MSCs-derived exosome improved functional behavioral recovery in the SCI animal model by shifting microglial polarization from M1 to M2 phenotype [120]. MiR-216a-5p down-regulated TLR4/NF-κB/PI3K/AKT signaling cascades, improved M2/M1 ratio, and thereby favored amelioration of traumatic SCI [120].

Similarly, another study indicated that intravenously injected MSCs-derived exosomes improved M2/M1 ratio, providing an anti-inflammatory environment in the injured spinal cord [121]. BM-MSCs-derived exosome also promoted the rescue of locomotor function and M2-phenotype polarization in a rat model of SCI [122]. Injected exosome suppressed neuronal apoptosis and degeneration and down-regulated inflammatory responses in treated rodents by miR-125a-mediated down-regulation of interferon regulatory factor 5 (IRF5) [122]. IRF5 is a transcription factor that contributes to the type I interferon and the TLRs inflammatory signaling axes [123]. It can also enhance macrophage polarization toward the M1-phenotype and stimulate neuronal destruction in pathological conditions. Thereby, exosomal miR-125a elicits neuroprotective impacts through down-regulation of IRF5 expression in SCI rats [122]. Likewise, Li and colleagues (2020) found that miR-124-3p containing BM-MSC-derived exosomes attenuated SCI and restored neurological dysfunction in a rat model by suppression of endoplasmic reticulum to nucleus signaling 1 (Ern1) protein. The Ern1 is involved in pro-inflammatory cytokine expression and stimulation of M1 polarization [124]. Thus, miR-124-3p may be a novel therapeutic target to alleviate SCI by suppressing Ern1 expression and increasing M2 polarization. In another study, given that the miR-29 family serves as an essential survival factor for neuronal cells, MSCs-derived exosomes were transduced post-isolation with miRNA-29b to evaluate their effect on SCI [125]. The systemic injection of miRNA-29b containing exosomes improved hind limb motor function, reduced histopathological damage, and induced neuronal regeneration in spinal cord tissues in SCI rodent models. Targeting the proteins complicated in neuronal regeneration, such as neurofilament-200 (NF200), growth-associated protein 43 (GAP-43), and glial fibrillary acidic protein (GFAP), was thought to be responsible for the favored effects upon exosome therapy in SCI rats [125]. Also, systemic injection of exosomes derived from genetically modified MSCs to overexpress miR-544 [126], miR-133b [127], and miR-126 [128] alleviated histologic deficits, ameliorated neuronal loss, down-regulated inflammation, stimulated neurogenesis, and finally improved hindlimb locomotor function in SCI rats.

In addition to inhibiting inflammatory responses, MSCs-derived exosomes can exert pro-angiogenic impacts on endothelial cells and thus stimulate angiogenesis in the SCI model, offering great capacity for SCI therapy [71, 129]. Meanwhile, local administration of human urine MSCs-derived exosomes embedded in hydrogel might improve SCI-induced dysfunction probably via improving angiogenesis through angiopoietin-like 3 (ANGPTL3) delivery [130]. Apart from playing a pivotal role in lipid metabolism, ANGPTL3 induces angiogenesis by improving the pro-angiogenic aptitude of endothelial progenitor cells (EPCs) and triggering ECs’ adhesion and migration through integrin αvβ3. These properties make it an ideal target for various CNS-related disorders [131]. Activation of autophagy [132] and improvement in the integrity of the blood–spinal cord barrier (BSCB) [133] are other relevant mechanisms by which MSCs-derived exosomes stimulate functional recovery in SCI rodents. Autophagy can attenuate neuronal injuries and ameliorate locomotor function by decreasing apoptosis following SCI [134]. As well, a significant association between promoted BSCB integrity and potentiated locomotor recovery has strongly been observed [135]. In a recent study, MSCs-derived exosomes provoked autophagosome formation, reduced cleaved caspase-3, and improved anti-apoptotic protein B cell lymphoma-2 (Bcl-2) levels in vitro and in the SCI rat model [132]. Moreover, these exosomes can decrease pericyte migration and thereby reduce the permeability of the BSCB by inhibiting NF-κB p65 signaling [133]. The MSCs-derived exosome also can support BSCB integrity by down-regulation of MMPs activity, thereby enhancing the functionality of cell junction proteins and reducing BSCB permeability [136]. These findings justify the MSCs-derived exosome application for SCI therapy.

Recent reports have revealed that UC-MSCs-derived exosome delivery using an alginate scaffold can induce neurite outgrowth, reduce inflammatory cytokine levels, and enhance IL-10 and GDNF levels in SCI rats [137]. Transplantation of exosomes immobilized in an adhesive hydrogel provoked nerve recovery and preserved urinary tissue by a marked inhibition of neuroinflammation and oxidation [138]. The hydrogel enables sustained release of embedded exosomes and thus can ease the long-term delivery of exosomes’ components to damaged tissue [138]. Likewise, implantation of BM-MSC-derived exosome encapsulated in gelatin methacrylate hydrogel (GelMA) enhanced exosomes’ retention, promoted neurogenesis, and diminished glial scars in the lesion sites in an SCI preclinical model [139].

### TBI

Traumatic brain injury (TBI) is a leading cause of death and chronic disability in young and elderly patients in industrialized and developing countries. It brings about fleeting or enduring physical, cognitive, and behavioral dysfunctions [140]. Upon TBI, various signaling molecules and metabolic instabilities, mainly impair BBB, induce neuroinflammation, cerebral edema, mitochondrial dysfunction, and oxidative injury [141].

In rats with controlled cortical impact (CCI)-induced TBI, systemic injection of MSCs-derived exosome reduced TBI severity and improved spatial learning and functional sensorimotor recovery [142]. In addition to the down-regulation of inflammation, the exosome therapy enhanced neonatal ECs in the lesion zone and mature neurons in the dentate gyrus (DG) [142]. Thereby, it was thought that stimulation of angiogenesis and neurogenesis and also inhibition of inflammatory responses play critical roles in exosome-mediated sensorimotor functional recovery and improved spatial learning in rats after TBI [104, 143]. Meanwhile, Zhang et al. (2015) revealed that exosome treatment strikingly augmented the vascular density in the injured cortex and DG in rats with TBI, as evidenced by an increase in newborn ECs [104]. In mice with CCI-induced TBI, exosomes administration by intraorbital route also diminished the lesion size and ameliorated neurobehavioral function [144]. Also, improvement in Bax/Bcl-2 ratio, reduction in TNF-α, IL-1β, and iNOS, and induction of M2 polarization signified the key role of pro-survival pathways and suppression of neuroinflammation in this regard [144]. Also, Chen and coworkers (2020) indicated that exosomes derived from AT-MSCs could inhibit microglia activation by suppression of NFκB and P38 MAPK signaling, thus reducing inflammation and enabling functional recovery [145].

Interestingly, down-regulation of the high-mobility group box 1 (HMGB1)/TLR4 pathway is believed to be responsible for functional recovery in rodents with brain injury following MSCs-derived exosome transplantation [23]. HMGB1 is a typical damage-associated molecular pattern (DAMP) protein and elicits its biological function predominantly via binding to TLR4 [146]. The connection between HMGB1 and TLR4 eventually leads to the progression of neuroinflammation and resultant neurodegeneration. Xiong et al. (2020) showed that although brain damage results in improved levels of HMGB1, TLR4, TNF-α, and p53, administration of the MSCs-derived exosome could reduce their levels in treated murine in part by miRNA129-5p delivery [23]. MiRNA129-5p mediates anti-inflammatory and anti-apoptotic effects by down-regulation of the HMGB1/TLR4 pathway [23]. The encouraging outcomes also were exhibited in larger animal models of TBI [90, 147]. A study in swine models of TBI demonstrated that systemic administration of MSCs-derived exosome could facilitate neuroprotection and support functional rescue by suppressing inflammation, as shown by reduced IL-1, IL-6, IL-8, and IL-18 [147]. Also, exosome therapy caused a reduction in NF-κB levels while improving BDNF levels in treated swine [147]. BDNF induces neuronal survival, synaptic plasticity, and neurogenesis in rats with TBI and thus holds great potential for applications in neurological disease therapy [148]. MSCs-derived exosome also attenuated cerebral edema (CE), which has an unfavorable prognosis in TBI. Exosome therapy also improved BBB integrity in swine with TBI via reducing albumin extravasation and improving laminin, Claudin-5, and ZO 1 levels [90]. As previously described, these proteins enable TJs between the ECs and thus support BBB integrity.

### Stroke

As the primary type of stroke, ischemic stroke is characterized by an early ischemic occasion that divests brain tissue from blood supply and oxygenation. Indeed, stroke yields permanent brain injury and succeeding motor and cognitive deficits [149]. Based on the literature, the inflammatory response of glial cells is the chief reason for brain injury during stroke [150]. In this condition, the recruitment of the immune cells to the ischemic zone in association with pathological mediators, such as oxidative stress, excitotoxicity, MMPs, HMGB1, TLR4, arachidonic acid metabolites, and MAPK, might spread ischemic brain injury [151]. Indeed, systemic and local immune responses contribute to the primary and secondary development of ischemic lesions. Thus, immune response affects repair, recovery, and overall outcome after a stroke [152]. Also, accumulating proofs display that autophagy is triggered in numerous cell types in the brain, including neurons and glial cells, following ischemic stroke [150, 153]. However, autophagy plays a double-edged sword role in stroke. Although autophagy may trigger neuroprotection upon stroke, its activation in some circumstances may result in cell death [152].

Upon stroke, reports have shown that CD14 receptors and TLR4 are robustly expressed in activated microglia in the infarct brain zone, enabling microglial activation [154]. These findings have outlined the importance of TLR4 expression in the proceeding of stroke-induced pathological events. Recent studies in middle cerebral artery occlusion (MCAO) mice, a standard cerebral stroke model, showed that exosomal miR-542-3p could decrease neuronal destruction, inhibit inflammatory microglial activation, and ultimately reduce infarct volume in vivo [155]. Indeed, MSCs-derived exosomes inhibited ischemia-induced glial cell inflammatory reactions by targeting TLR4, thereby exerting neuroprotective effects in vivo [155]. Furthermore, miR-25-3p containing AT-MSCs-derived EVs reduced autophagy and the size of the infarct cavity and eventually elicited neurological recovery in a mice model of stroke [156]. In vitro analysis revealed that AT-MSC-derived EVs constrained autophagy through down-regulation of p53 and B cell lymphoma 2-interacting protein 3 (BNIP3) [156]. The p53 and BNIP3 are two primary positive regulators of autophagy, and their protein levels are usually enhanced in the infarct region of mice after stroke [156]. Similarly, exosomes from miR-30d-5p-overexpressing AT-MSCs also decreased infarct volume by inhibiting autophagy concomitant with triggering M2 microglia polarization [157]. The potent effects of the MSCs-derived exosome on the determination of microglial fate have been evidenced by other studies [158, 159]. For instance, miR-146a [158], miR-146a-5p [159], and miR-223-3p [160]-enriched exosomes derived from MSCs could ameliorate neurological activities, attenuate apoptotic neurons, and deter neuroinflammation most probably by inhibition of M1 polarization in rats with ischemic stroke. The miR-146a containing exosome reduced iNOS, cyclooxygenase-2 (COX-2), and monocyte chemoattractant protein-1 (MCP-1) expression and simultaneously suppressed activation of IL-1 receptor-associated kinase1 (IRAK1) and nuclear factor of activated T cells 5 (NFAT5) [158]. Likewise, exosomal miR-146a-5p attenuated microglial-induced neuroinflammation by negative regulation of IRAK1/TRAF6 signaling pathway in a rodent model of stroke [159]. IRAK1/TRAF6 pathway serves essential roles in signal transduction of the TLR/IL-1R superfamily, thereby targeting this axis averts inflammatory responses and inspires neuroprotection [159]. Exosome treatment also suppressed microglial M1 polarization by down-regulation of cysteinyl leukotriene two receptors (CysLT2R). These well-known inflammatory receptors mainly participate in inflammation and neuronal injury by stimulating microglia M1 polarization [160]. In addition to their capability to reduce neuroinflammation mainly via inhibiting M1 microglial-induced neuroinflammation and targeting key inflammatory signaling axes, MSCs-derived exosomes could also ameliorate functional recovery in animal models of stroke by improving the neuroplasticity [71, 161]. Meanwhile, Xin et al. (2017) implied that systemic injection of miR-17-92 cluster-enriched exosome derived from MSCs could improve ODCs, neurogenesis, and neurite remodeling/neuronal dendrite plasticity in the ischemic boundary zone (IBZ) in MACO rats [161]. These desired effects were elicited by targeting phosphatase and tensin homolog (PTEN) by miR-17-92, which in turn stimulates PI3K/Akt/mechanistic target of rapamycin (mTOR)/glycogen synthase kinase (GSK) 3β signaling pathway [161]. Based on previous reports, activation of this pathway exceeds cell survival and neuroplasticity and could promote functional recovery in neurological conditions [152, 162]. Also, exosomes from MSCs might transport miR-133b to neurons and astrocytes, thus provoking neurite remodeling and functional rescue in rodents after stroke [163].

A summary of main reports depending on exosome treatment in animal models of acute neurodegeneration is provided in Table 1.

**Table 1:** MSCs-derived secretome (e.g., exosome) therapy in preclinical models of acute neurodegeneration

Condition	Model	Cell source	Administration route	Results	Ref.
SCI	Mice	BM	Intravenous	Stimulating functional behavioral restoration via improving M2/M1 macrophage ratio by exosomal miR-216a-5p	[120]
SCI	Rat	AM	Intravenous	Reducing inflammation and eliciting antioxidant effects through MSC-derived exosomes immobilized in hydrogel	[138]
SCI	Rat	UC	Intrathecal	Plummeting pro-inflammatory cytokine TNF-α and IL-1β, while improving IL-10, BDNF, and GDNF levels in brain tissue	[164]
SCI	Rat	BM	Intravenous	Alleviating neurological damage through suppressing Ern1 and promoting M2 macrophage by exosomal microRNA-124-3p	[165]
SCI	Rat	BM	Intravenous	Repairing spinal cord injury by exosomal miRNA-29b	[166]
SCI	Rat	BM	Intravenous	Repairing spinal cord injury by inhibition of A1 neurotoxic reactive astrocytes activation	[117]
SCI	Rat	UC	Intrathecal	Reducing the c-Fos, GFAP, Iba1, TNF-α, and IL-1β, and improving IL-10 and GDNF levels	[137]
SCI	Rat	BM	Intravenous	Inhibition of pericytes migration and improving the BSCB integrity by targeting NF-κB p65 signaling in pericytes	[133]
SCI	Mice	BM	Intraspinal	Promoting angiogenesis and axon growth leading to the functional rescue	[167]
SCI	Rat	BM	Intravenous	Promoting the neurogenesis and angiogenesis, while reducing apoptosis by exosomal miR-126	[128]
SCI	Rat	BM	Intravenous	Hindrance of complement activation	[168]
SCI	Mice	Placental	Intrathecal	Potentiating angiogenesis leading to the ameliorated neurologic function	[129]
SCI	Rat	BM	Intravenous	Improving M2 macrophage polarization	[121]
SCI	Rat	UC	Intravenous	Exerting anti-inflammatory and anti-fibrotic action	[169]
SCI	Rat	NA	Intravenous	Attenuation of inflammation through down-regulation of TLR4/NF-κB signaling pathway by exosomal miR-145-5p	[170]
SCI	Rat	BM	Intravenous	Inhibition of A1 neurotoxic reactive astrocytes activation in part via suppressing NF-κB translocation	[118]
TBI	Rat	BM	Intravenous	Promoting functional recovery through triggering endogenous angiogenesis and neurogenesis and also down-regulation of neuroinflammation	[104]
TBI	Rat	AT	Intracerebroventricular	Reducing microglia activation	[145]
TBI	Rat	BM	Intravenous	Amelioration of sensorimotor and cognitive dysfunction, attenuation of hippocampal neuronal cell loss, inducing the angiogenesis and neurogenesis, and mitigation of neuroinflammation	[143]
TBI	Rat	BM	Intranasal	Exosome efficient migration to the injured Forebrain	[171]
TBI	Rat	BM	Intravenous	Amelioration of neurological functions, decreasing brain edema through favoring BBB integrity	[23]
TBI	Swine	BM	Intravenous	Boosting neural plasticity along with reducing inflammation and apoptosis ensuring reduced brain lesion zone	[147]
TBI	Mice	BM	Intraorbital	Mitigation of early inflammatory responses	[144]
TBI	Rat	BM	Intravenous	Triggering endogenous angiogenesis and neurogenesis	[142]
TBI	Swine	BM	Intravenous	Induction of neuroprotection and supporting BBB integrity	[90]
TBI	Rat	BM	Intravenous	Stimulating neuroprotection via exosomal miR-216a-5p, which up-regulates BDNF expression	[172]
TBI	Mice	BM	Intravenous	Amelioration of cognitive deficits	[173]
Stroke	Mice	AT	Intravenous	Reducing autophagy by exosomal miR-25	[174]
Stroke	Rat	BM	Intravenous	Stimulation of neuritis outgrowth by exosomal miR-133b	[163]
Stroke	Mice	BM	Intravenous	Obstruction of neuroinflammation and averting cerebral infarction by exosomal miR-542-3p	[175]
Stroke	Mice	UC	Intravenous	Attenuation of microglial-mediated neuroinflammation through down-regulation of the IRAK1/TRAF6 signaling axis by exosomal miR-146a-5p	[159]
Stroke	Rat	BM	Intravenous	Promoting the neurorestorative effects	[176]
Stroke	Rat	BM	Intravenous	Suppression of the neuronal apoptosis and M1 macrophage polarization by exosomal miR-146a-5p causes alleviated intracerebral hemorrhage	[177]
Stroke	Mice	BM	Intravenous	Induction of neuroprotection	[178]
Stroke	Rat	BM	Intravenous	Improving the functional recovery and neurovascular plasticity	[179]
Stroke	Rat	UCB	Intravenous	Triggering the functional recovery	[180]
Stroke	Rat	AT	Intravenous	Attenuation of the ischemic brain injuries by targeting miR-21-3p/MAT2B axis	[181]
Stroke	Rat	BM	Intravenous	Promoting the axon–myelin remodeling by exosomal miR-17-92	[182]
Stroke	Mice	BM	Intravenous	Reducing inflammation, pathological alterations and apoptosis by exosomal miR-221-3p	[183]

*MSCs* mesenchymal stromal cells, *TBI* traumatic brain injury, *SCI* spinal cord injury, *AT* adipose tissue, *BM* bone marrow, *UCB* umbilical cord (UC) blood, *miRNAs* microRNAs, *IL* interleukin, *TNFα* tumor necrosis factor α, *BSCB* blood–spinal cord barrier, *GDNF* glial cell-derived neurotrophic factor, *NF-κB* nuclear factor kappa B, *TLRs* toll-like receptors, *BBB* blood–brain barrier, *TRAF6* TNF receptor-associated factor 6, *IRAK1* interleukin 1 receptor-associated kinase 1, *BDNF* brain-derived neurotrophic factor, *NA* not applicable

## MSCs-derived exosome in chronic neurodegeneration

### MS

Multiple sclerosis (MS), a common CNS degenerative disorder, is characterized by the degradation of myelin proteins as a result of the dysregulated immune response. Indeed, MS is a heterogeneous, multifactorial, immune-related condition that establishes demyelinating brain and spinal cord lesions, correlating with neuro-axonal injuries [184, 185]. Focal lesions are mainly induced by infiltrating immune cells, such as *T* cells, *B* cells, and myeloid cells, into the CNS parenchyma. Deregulation in the M1/M2 microglia ratio and irregular NLRP3 inflammasome activations also contributes to the pathogenesis of MS [186]. In an early MS lesion, the primary pool of phagocytic cells consists of 40% microglia [187]. Thereby, deterring inflammation and provoking remyelination are central therapeutic goals in this condition.

Recent reports have exposed that UC-MSCs-derived exosomes could avert the proliferation of peripheral blood mononuclear cell (PBMC)-derived from MS patients in vitro, offering new opportunities to alleviate MS severity [188]. Studies in experimental autoimmune encephalomyelitis (EAE) mice, the most common model of MS, also have exhibited the therapeutic potential of exosome by secretion of immunomodulatory factors, down-regulation of NALP3 inflammasome activation, and also deterring of NF-κB expression levels in vivo [189]. The down-regulation of the NF-κB canonical pathway reduces EAE pathology. Conversely, knocking out NF-κB regulatory protein A20 in microglia might trigger microglial activation, neuroinflammation, and enhancement of EAE pathology [190]. Accordingly, it seems that NF-κB can be a rational target for MS therapy. Transplantation of MSCs-derived exosomes also improved the levels of M2-related cytokines such as IL-10 and TGF-β levels and decreased the levels of M1-related TNF-α and IL-12 in EAE mice, thus attenuating inflammation and demyelination of the CNS [191]. Based on the previous findings, TGF-β1 enhances remyelination and reduces disease severity in EAE by promoting ODCs maturation and resultant remyelination [192]. Also, low IL-10 production is related to the higher disability and MRI lesion load in secondary progressive MS patients. Hence, improved levels of IL-10, as shown in post-exosome treatment, may offer desired outcomes in MS patients [193].

Further, growing information has demonstrated that IL-37, as a member of the IL-1 family, could induce beneficial effects in MS due to its potent anti-inflammatory influences [194]. Notably, IL-37 may be part of a feedback loop to regulate inflammation in MS pathogenesis. Further, IL-37 overexpressing mice also exhibit substantial resistance versus functional deficits and demyelination upon MS and SCI [195]. Injection of secretome derived from hypoxia-preconditioned human periodontal ligament (hPL) MSCs into C57BL/6 mice with EAE also resulted in decreased clinical and histologic disease scores mainly through anti-inflammatory cytokine IL-37 [196]. Also, intervention alleviated oxidative stress and autophagic and apoptotic markers in treated mice [196]. Recent reports have displayed that IL-37 serves a critical role in averting innate and adaptive immune reactions and inflammatory responses in MS patients [194, 196, 197]. Besides, another study showed that systemic injection of MSCs-derived exosome promoted cognitive function, enhanced newly generated ODCs numbers, up-regulated myelin essential protein (MBP) levels, and finally diminished neuroinflammation by promoting M2/M1 microglia ratio in a rodent model of MS [198]. The exosome-mediated anti-inflammatory effects probably relied on the down-regulation of the TLR2/IRAK1/NFκB pathway post-transplantation [198]. This axis plays a pathological role in MS progress by impairing BBB integrity and aberrant T cells and B cells activation [199].

Interestingly, Reynolds et al. (2010) have suggested that TLR2 deficiency in Th17 cells may attenuate their ability to inspire EAE [200]. Further, neural cell proliferation and remyelination of axons following treatment with MSCs-derived secretome were found to be underlying by hepatocyte growth factor (HGF) and its primary receptor cMet in EAE mice [201]. The HGF is a pleiotropic cytokine with substantial anti-inflammatory possessions. It hinders both the generation and action of cytotoxic *T* lymphocytes (CTLs) from naïve CD8 + T cells and inhibits CD4 + T cell CNS autoimmunity in MS preclinical models [202]. In addition to MSCs-derived exosome, intranasal administration of the exosome isolated from MSCs-differentiated ODCs could reduce pathological symptoms in EAE mice [203]. Analysis indicates that BDNF, GDNF, and ciliary neurotrophic factor (CNTF) delivery concomitant with boosted remyelination, as evidenced by evaluating MBP and ODCs transcription factor levels, play a key role in this regard [203].

### AD

Alzheimer’s disease (AD) is described by extracellular aggregates of amyloid β (Aβ) plaques and intracellular neurofibrillary tangles (NFTs) created by hyperphosphorylated τ-protein in the brains’ cortical and limbic regions [204, 205]. Such aggregates inspire cytotoxicity versus neurons by stimulating pore formation, which leads to the leakage of ions, disturbance of cellular calcium levels, and impairment of membrane potential [206]. These events instigate apoptosis, synaptic loss, and cytoskeleton impairments and finally yield cognitive, learning, behavioral, and motor dysfunctions [207]. Concerning the biochemical and neuropathological studies, microglia are infiltrated and then activated to meet the clearance of Aβ. The continual activation of the microglia and other immune cells potentiates neuroinflammation and facilitates AD progress [208].

In 2021, Chen et al. showed that systemic injection of 50 μg of purified Wharton’s jelly (WJ)-MSC-derived exosomes promoted neuronal memory/synaptic plasticity and reduced cognitive deficits in AD mice. It was found that these effects were mediated by down-regulation of histone deacetylase 4 (HDAC4), a negative regulator of neural plasticity gene expression [209]. However, they indicated that the injected dose of exosomes was not sufficient to degrade the aggregates in treated mice [209]. Also, exosome treatment caused a pro-survival effect on neuronal cells by reducing the Bax/Bcl-2 ratio, inactivating cleaved caspase-3, and restoring mitochondrial dysfunctions in the AD in vitro model [210]. Notably, the intervention led to diminished Aβ40 and Aβ42 levels and induced neuritis growth in the AD mice model, making them a valued therapeutic source to down-regulate Aβ-inspired neuronal loss in AD [210]. Another study also indicated that AT-MSCs-derived exosomes include active neprilysin (NEP), the chief Aβ-degrading enzyme in the brain [211]. These exosomes thus could stimulate Aβ degradation and consequently reduce related neural loss post-transplantation [211]. Mice with NEP deficiency display impairment in spatial working memory, suffer from astrocytosis, and show an enhanced level of soluble Aβ42 and extracellular Aβ deposition [212]. Another report also implied that hUCB-MSCs could diminish Aβ42-induced synaptic deficits by potentiating thrombospondin-1 (TSP-1) secretion, thereby offering a capable alternative therapeutic strategy for early-stage AD [213]. TSP-1 is secreted typically by astrocytes and serves as a modulator of synaptogenesis and neurogenesis. Its expression is diminished in AD brains; however, TSP-1 exogenous administration into AD model mouse brains may attenuate the destructive effects of Aβ on synaptic proteins [214]. In vivo, the injection of TSP-1 enriched hUCB-MSCs-derived secretome up-regulated the expression of synaptic density markers, such as synaptophysin (SYP) and post-synaptic density protein-95 (PSD-95), in hippocampal neurons of Aβ42-treated mice [213]. Indeed, TSP-1 could induce the expression of α2δ-1, a voltage-activated Ca2 + channel subunit, and the synaptic adhesion molecule neuroligin-1 (NLGN1), leading to potentiated synaptogenesis in animal models [213]. Neuroligins adjust synapse formation and function, and down-regulation of their activation results in synaptic and memory deficits, as shown in AD mice [215, 216]. Thereby, restoration of their expression and function as facilitated by administration of MSCs-derived exosome may attenuate AD-associated pathological symptoms. On the other hand, it appears that iNOS may serve as the initiator of Aβ deposition and AD progression [217]. There is clear evidence verifying the correlation between improved iNOS levels with cerebral plaque creation, astrocytosis, and microgliosis [218]. Wang et al. (2018) displayed that intracerebroventricular administration of MSC-derived exosome potently reduced iNOS expression [219]. As a result, intervention ameliorated cognitive behavior and supported synaptic transmission of hippocampal CA1 neurons in APP/PS1 mice [219]. Besides, secretion of GDF-15 at measurable levels has strongly been proved from MSCs. Normally, GDF-15 can be secreted by damaged neurons and microglial cells, mainly contributing to the Aβ clearance capacity of microglial cells [220]. Interestingly, Kim and coworkers (2018) showed that administration of GDF-15 containing UCB-MSCs-derived exosome reduced Aβ plaques in the brains of 5XFAD mice [221]. Molecular analysis exhibited that GDF-15 could increase the expression of insulin-degrading enzyme (IDE) in microglial cells and thereby heighten their capacity to degrade Aβ plaques [221]. IDE act as the primary regulator of Aβ levels in neuronal and microglial cells. IDE − / − mice experience enhanced cerebral plaques of endogenous Aβ [222]. As a result, exosomal GDF-15 up-regulates IDE activations and consequently attenuates neural loss in treated AD mice. Likewise, GDF-15-enriched BM-MSCs-derived exosomes intensified NEP and IDE expression by inducing AKT/GSK-3β/β-catenin pathway. Enhanced NEP and IDE expression results in degrading Aβ42 protein in treated AD rodents [220]. These two reports confer an encouraging therapeutic plan for AD by targeting proteins complicated in Aβ degradation.

Apart from the cited biomolecules, miRNA contents of MSCs-derived exosomes play essential roles in electing favored outcomes post-transplantation in AD preclinical models. For example, miR-223 containing exosomes reduced neural cell loss in an AD in vitro model by suppressing PTEN expression and thus stimulating PI3K/Akt pathway. PI3K/Akt pathway plays a crucial role in neuroprotection, increasing cell survival by inspiring cell proliferation and delaying apoptosis [28]. Also, several reports have shown that exosomal miR-146a [223] and miR-29 [224] secreted by MSCs could suppress NF-κB pathways. Down-regulation of NF-κB pathways inhibits the expression of the various pro-inflammatory cytokine and consequently reduces Aβ-mediated cytotoxicity in AD murine models [224].

### PD

Parkinson's disease (PD) is characterized by motor symptoms like tremor, rigidity, slowness of movement, gait problems, fatigue, depression, pain, and cognitive deficits [225]. Dopaminergic (DA) neuron loss in the substantia nigra (SN), reduction in striatal dopamine levels, and intracellular aggregates of α-synuclein are the chief PD neuropathological hallmarks [226]. The corresponding molecular pathogenesis comprises various pathways and mechanisms: α-synuclein proteostasis, mitochondrial dysfunctions, aberrant oxidative stress, deregulated calcium homeostasis, impaired axonal transport, and neuroinflammation [227]. Recent results in the 6-hydroxydpomanie (6-OHDA) rat PD model revealed that intrastriatal administration of BM-MSCs-derived secretome could ameliorate motor behavior and increase DA neurons in SN and fibers in the striatum [101]. In vitro, BM-MSCs-derived secretome also improved the differentiated neurons frequency, as evidenced by enhancement in MAP-2 staining [101]. Another study in a Caenorhabditis elegans model of PD showed that BM-MSCs-derived exosome could markedly reduce α-syn-induced DA neuron loss [228]. Interestingly, in silico investigations verified the presence of potent suppressors of α-syn proteotoxicity, such as BDNF and VEGF-B, in these exosomes [228]. Such growth factors intensify the effects of peroxisome proliferator-activated receptor-γ coactivator 1-α (PGC1α), which acts as a neuroprotective factor and alleviates the damaging effects of a-syn on neuronal cells [229]. VEGF improves neuroprotection indirectly through induction of the proliferation of glia and triggering angiogenesis in PD experimental models [230]. Besides, BDNF improves the survival of DA neurons and sustains dopaminergic neurotransmission and motor function [231]. In other studies, neurobehavioral deficits, neuroinflammation, oxidative stress, and neural cell apoptosis were alleviated in the 6-OHDA murine model of PD following injections of MSCs-derived exosome [232]. Further studies to clarify the corresponding mechanism behind the exosome-elicited anti-inflammatory and pro-survival effects on the PD animal model have conferred the key role of miR-188-3p [233]. The exosomal miR-188-3p derived from MSCs could inhibit both cyclin-dependent kinase 5 (CDK5)-mediated autophagy and NLRP3-mediated inflammation in PD rodent models, thus inducing neuroprotection against PD-associated toxicities [233]. Exosomes also mediate antioxidant effects by the transportation of the mitochondrial NAD-dependent deacetylase sirtuin-3 (SIRT3) [234]. The SIRT3 largely contributes to adjusting mitochondrial quality control in neuronal mitochondria [235]. It constrains degeneration of DA neurons and corrects behavioral abnormalities by increasing the functional potential of mitochondria [236]. As described, mitochondrial dysfunction in the DA neurons is a common pathological event observed in PD patients. The mitochondrial dysfunction is mainly characterized by ROS generation, decreased activity of mitochondrial complex I enzyme, enhanced cytochrome-c release, ATP exhaustion, and caspase-3 activation [237, 238]. These events, in turn, lead to DA degeneration. Significantly, SIRT3 alleviates oxidative stress-mediated damages by inducing several antioxidant factors, such as forkhead box O3 (FOXO3), and superoxide dismutase (SOD) [239]. Thereby, SIRT3 likely can be an effective disease-modifying approach for PD patients.

In addition to the induction of neurogenesis and neuroprotection, MSCs-derived exosome was found to enable the recovery of PD by promoting intracellular adhesion molecule-1 (ICAM1)-mediated angiogenesis of human brain microvascular endothelial cells (HBMECs) in a mice model of PD [240]. HBMECs are a dominant component of the microvasculature that shape the BBB and defense the brain versus toxins and immune cells by paracellular, transcellular, transporter, and ECM proteins [241]. Potentiated angiogenesis of HBMECs following exosome therapy might be attributable to activating the SMAD family member 3 (SMAD3) and P38MAPK axis [240]. Chen et al. (2020) also demonstrated that inducing autophagy is another tool by which MSCs-derived exosome facilitates the amelioration of apomorphine-induced asymmetric rotation [242]. Exosome-mediated autophagy also could reduce DA neuron loss in SN and improve dopamine levels in the striatum of the PD rodent model [242]. In vitro, exosome could increase the level of 6-OHDA-induced SH-SY5Y cell autophagy, as documented by promoted expression of LC3B-II/I and Beclin-1 [242]. As neuronal autophagy is the primary process for the degradation of an abnormal protein aggregate [243, 244], it appears that exosome treatment stimulates neuroprotection against toxic proteins by stimulating the autophagy process in neuronal cells. But, other reports suggest that dysregulation of autophagy inspires the accumulation of abnormal proteins and/or damaged organelles [142]. Further studies are required to clarify the therapeutic values of targeting autophagy for neurological disease therapy.

### ALS

Amyotrophic lateral sclerosis (ALS) is typically characterized by the destruction of the large pyramidal neurons in the motor cortex and related corticospinal tracts [245, 246]. Of course, lower motor neurons (MNs) are also damaged during this disease. The clinical appearances of ALS show death of both upper and lower MNs with muscle denervation [247]. Although ALS etiology is still partially elucidated, that mutation in the Cu/Zn superoxide dismutase SOD1 gene is shown in about 20% of patients with familial ALS [248]. Current clinical trials have evidenced the safety and modest efficacy of MSCs administration in ALS patients, which is mainly achieved by suppressing neuroinflammation and improving MNs survival [249–251].

In 2018, Bonafede et al. indicated the neuroprotective role of AT-MSCs-derived exosome in an in vitro model of ALS [252]. The analysis revealed suppression of pro-apoptotic proteins Bax and cleaved caspase-3 expression in association with improved anti-apoptotic protein Bcl-2 expression in treated models post-transplantation [252]. These alterations in the expression profile of apoptosis-associated protein reflect the strong potential of MSCs-derived exosomes to deliver pro-survival effects on target MNs, thus favoring neuroprotection [252]. Likewise, AT-MSCs-derived exosomes (0.2 µg/ml) protected NSC-34 cells from hydrogen peroxide-induced oxidative damage, which is suggested as the dominant mechanism of injury in ALS [253]. The observed effects could potently be induced by miRNA21, miRNA222, and miRNAlet7a, which have previously been identified in MSCs-derived exosomes. These miRNAs act as negative regulators of apoptosis as well as an inducer of proliferation [254]. MSC-derived conditioned medium (CM) also could up-regulate the expression of neurotrophic factors (e.g., GDNF and CNTF) in astrocytes and VEGF in NSC-34 cells [255]. GDNF possesses a high affinity for MNs and can avert their death and prohibit muscle atrophy upon many insults [256]. The connection of GDNF to its receptors, in turn, activates multiple intracellular signaling axes and leads to supporting the development and preservation of neuron–neuron and neuron–target tissue interactions [257]. Thus, it appears that MSCs-derived exosomes moderate MN and glial response to apoptosis and inflammation, proposing them a preferred therapeutic strategy to treat ALS [255, 258, 259]. As well, another study in SOD1^G93A^ mice, the most common ALS animal model, also exposed the efficient potential of exosome to treat ALS [260]. Upon intravenous and intranasal administration, implanted exosomes were capable of restoring motor function, protecting lumbar MNs and neuromuscular junction (NMJ) accompanied by mitigation of glial cells activation in vivo [260]. These results provide further knowledge for the capable application of MSCs-derived exosome in ALS patients.

A summary of main reports depending on exosome treatment in animal models of chronic neurodegeneration is provided in Table 2.

**Table 2:** MSCs-derived secretome (e.g., exosome) therapy in preclinical models of chronic neurodegenerative diseases

Condition	Model	Cell source	Administration route	Results	Ref
AD	Mice	BM	Intranasal	Stimulation of neuroprotection in part through inhibition of neuroinflammation	[99]
AD	Mice	BM	Intravenous	Attenuation of Aβ levels and provoking anti-inflammatory impact leading to the amelioration of learning and memory functions	[261]
AD	Mice	BM	Intravenous	Reducing cognitive deficits through exosomal miR-146a	[262]
AD	Rat	BM	Intravenous	Induction of neurogenesis	[263]
MS	Mice	PDL	Intravenous	Inhibition of NLRP3 inflammasome activation	[189]
MS	Mice	BM	Intravenous	Attenuation of demyelination lesion area and reducing diseases severity	[186]
MS	Mice	BM	Intravenous	Triggering remyelination and attenuation of the neuroinflammation	[198]
MS	Mice	BM	Intravenous	Reducing diseases severity by HGF delivery	[201]
MS	Mice	AT	Intravenous	Improving the Tregs population and IL-4 levels	[264]
MS	Mice	PDL	Intravenous	Improving IL-37 expression causing down-regulation of the pro-inflammatory cytokines levels	[265]
MS	Mice	SHEDs	Intravenous	Attenuation of demyelination and axonal injury, inhibition of inflammatory cell infiltration and also promoting M2/M1 macrophage ratio	[266]
PD	Rat	BM	Intravenous	Reducing the deterioration of DA neuron in SN and thus promoting dopamine levels in striatum	[100]
PD	Rat	BM	Intravenous	Induction of the protective effect on DA neuron	[101]
PD	Mice	AT	Intravenous	Mitigation of autophagy and pyroptosis	[233]
PD	Mice	AT	Intraperitoneal	Eliciting the HBMECs angiogenesis	[102]
PD	Rat	AT	Intravenous	Exerting antioxidant effects by improving sirtuin 3 levels	[267]
PD	Rat	BM	Intravenous	Amelioration of motor activities	[268]
PD	Rat	BM	Intranigral Intrastriatal	Promoting neural plasticity	[269]
PD	C. elegans	BM	NA	Decreasing α-syn aggregates in striatum	[228]
HD	Mice	AM	Intraperitoneal	Amelioration of motor functions	[270]
ALS	Mice	AT	Intravenous Intranasal	Down-regulation of glial cells function, favoring motor neurons, and eliciting protective effect on neuromuscular junctions (NMJs)	[271]

*MSCs* mesenchymal stromal cells, *AD* Alzheimer’s disease, *PD* Parkinson’s disease, *ALS* amyotrophic lateral sclerosis, *HD* Huntington’s disease, *MS* multiple sclerosis, *BM* bone marrow, *AT* adipose tissue, *PDL* periodontal ligament, *Aβ* amyloid beta-peptide, *AM* amniotic membrane, *NLRP3* NLR family pyrin domain containing 3, *HBMECs* human brain microvascular endothelial cells, *FOXP3* forkhead box P3, *α-syn* alpha-synuclein, *SHED* stem cells from human exfoliated deciduous teeth, *NA* not applicable

## Conclusion and future directions

Although various clinical trials based on the application of naive MSCs for neurological disease therapy have been conducted or are ongoing, MSCs-derived exosome therapy has become a promising approach for treating neurological diseases. Growing experimental/clinical proofs imply that MSC-derived exosomes may become novel cell-free therapy agents with encouraging superiority over MSCs, such as no risk of tumor formation and low immunogenicity. Exosome also exhibits the better capability to convey therapeutic biomolecules. They mediate intercellular communication by conveying biologically active cargo to target cells in both physiological and pathological circumstances. In the context of emerging therapeutics in neurological disease, exosomes can be loaded with multiple cargoes to modify gene expression and protein activities in recipient cells. They may ultimately result in immunomodulation, angiogenesis, neurogenesis, neuroprotection, and degradation of protein inclusions. Albeit, given the limited quantity of procured exosomes, progress in exosomes’ isolation and designing novel approaches to acquiring a higher amount of exosomes is urgently required. In this light, it has been revealed that parental MSCs’ expansion in hollow fiber three-dimensional (3D) culture system [217, 218] or their seeding on biomaterial like 45S5 Bioglass^®^ (BG) [219, 220] or Avitene Ultrafoam collagen hemostat might enable the release of exosome at higher levels [221].

MSCs-derived exosomes have some disadvantages compared to MSCs. The lack of standard isolation and purification protocol and rapid clearance from blood after administration in vivo is the most critical drawback [272]. As the conventional isolated approaches mainly rely on density and size, some substances like lipoproteins and viruses may overlap with their features, leading to incomplete removal. To improve the progress of effective biomarkers for exosomes, sensitive, accurate, and rapid quantitative means are needed. Additionally, designing dependable potency tests to determine the therapeutic effects of MSCs-derived exosomes accompanied by defining the optimized administration route and doses is of paramount importance. Also, electing a more appropriate cell source is critical because the therapeutic benefits of MSC-derived exosomes, such as improving neuritis growth, may in part differ depending on the origin of MSCs [273]. As well, obesity attenuates the anti-inflammatory impacts of human AT-MSCs, challenging their application in neuroinflammation-related neurological diseases [274]. Finally, donor demographics can be predominantly significant when ascertaining proper stem cells for treatment.

## Data Availability

Not applicable.

## Abbreviations

MSCs: Mesenchymal stromal cells
TBI: Traumatic brain injury
SCI: Spinal cord injury
AD: Alzheimer’s disease
PD: Parkinson’s disease
ALS: Amyotrophic lateral sclerosis
HD: Huntington’s disease
MS: Multiple sclerosis
AT: Adipose tissue
BM: Bone marrow
UC: Umbilical cord
miRNAs: MicroRNAs
TNFα: Tumor necrosis factor-α
TGF-β: Transforming growth factor-beta
TSG6: TNFα-stimulated gene-6
GDNF: Glial cell-derived neurotrophic factor
NF-kB: Nuclear factor kappa B
TLRs: Toll-like receptors
BBB: Blood–brain barrier
BDNF: Brain-derived neurotrophic factor
